# Interleukin-1β Promotes Schwann Cells De-Differentiation in Wallerian Degeneration via the c-JUN/AP-1 Pathway

**DOI:** 10.3389/fncel.2019.00304

**Published:** 2019-07-09

**Authors:** Gang Chen, Xiaohe Luo, Wenjin Wang, Yimei Wang, Fei Zhu, Wei Wang

**Affiliations:** ^1^Department of Plastic and Reconstructive Surgery, Shanghai Ninth People’s Hospital, Shanghai Jiao Tong University School of Medicine, Shanghai, China; ^2^Department of Plastic Surgery, First Affiliated Hospital of Anhui Medical University, Hefei, China; ^3^Department of Plastic Surgery, First Affiliated Hospital of Nanchang University, Nanchang, China

**Keywords:** Wallerian degeneration, Schwann cells, IL-1β, MPZ, p75NTR, c-JUN/AP-1, de-differentiation

## Abstract

Schwann cells (SCs) de-differentiate in Wallerian degeneration (WD) following nerve injury and, by doing so, can actively promote nerve repair and functional recovery. An innate-immune response is an important component of the complex of events referred to as WD. Damaged peripheral nervous system SCs produce IL-1β and other inflammatory cytokines. We hypothesized that, in addition to a role in immune responses, IL-1β participates in de-differentiation and proliferation of SCs. qPCR and ELISA demonstrated that expression of IL-1β mRNAs and protein increased after nerve injury. Immunofluorescent staining and western blotting demonstrated that expression of the p75 neurotrophin receptor (p75NTR) was significantly increased and levels of myelin protein zero (MPZ) were significantly decreased after IL-1β exposure compared with control groups *in vitro* WD. Additionally, qPCR demonstrated that IL-1β elevated expression of the de-differentiation gene p75NTR and decreased expression of myelination locus MPZ and promoted SCs de-differentiation. Furthermore, immunofluorescent staining, western blotting, qPCR and ELISA revealed that IL-1β promoted c-JUN expression and activation of AP-1 activity of SCs in an *in vitro* WD model. Finally, Immunofluorescent staining illustrated that IL-1β elevated expression of Ki67 in SCs nuclei, the apoptosis of SCs were detected by TUNEL. SCs of WD produce IL-1β which promotes SCs de-differentiation and proliferation.

## Introduction

The ultimate goal of regenerative medicine research is to enable replacement of lost or damaged tissues or organs. Regeneration can potentially be accomplished using the processes of de-differentiation, trans-differentiation or reprogramming. Humans have a limited capacity to regenerate tissues or organs, including liver and the peripheral nervous system (PNS). These tissues respond to injury through cellular reprogramming, producing cells that specifically promote repair and regeneration (Jessen and Arthur-Farraj, 2019). In some cases, the process of regeneration involves de-differentiation of mature cells. De-differentiation is a mechanism in which terminally differentiated cells revert to a less-differentiated stage within the same lineage and allows cells to proliferate before re-differentiating, leading to the replacement of lost cells (Jopling et al., 2011).

Schwann cells (SCs), myelinated glial cells of the PNS, de-differentiate and convert to denervated SCs in Wallerian degeneration (WD) following nerve injury and, by doing so, can actively promote nerve repair and functional recovery (Jessen and Mirsky, 2008; Tricaud and Park, 2017) So, it is also described as repair SCs. Meanwhile, they activate a series of repair-related phenotypes (Jessen and Mirsky, 2016; Jessen and Arthur-Farraj, 2019). Following de-differentiation, SCs clear myelin debris by autophagy, in addition, SCs contribute to macrophage-mediated myelin removal and re-enter the cell cycle, proliferate, and then form bands of Büngner, which support and direct outgrowing axons to sites of innervation (Martini et al., 2008; Rotshenker, 2011; Brosius et al., 2017). Moreover, these cells express and secrete a large number of axonal growth promoting factors, then re-differentiate and myelinate regenerated axons, which eventually leads to substantial functional recovery (Woszczycka-Korczynska et al., 2013; Jessen and Mirsky, 2016). Damaged SCs may induce cell apoptosis and limit functional recovery of peripheral nerves (Zhao et al., 2017). This sequence emphasizes the central function of SC de-differentiation in PNS regeneration.

An innate-immune response is an important component of the complex of events referred to as WD. PNS injury induces immune and non-immune cells to produce cytokines and develop an efficient cytokine network during WD (Rotshenker et al., 1992; Be′Eri et al., 1998; Shamash et al., 2002). Before macrophage recruitment, injured PNS produce IL-1β and other inflammatory cytokines (Rotshenker, 2011). These inflammatory cytokines have an irreplaceable effect on the initiation and regulation of inflammation during injury (Bastien and Lacroix, 2014). Certain inflammatory cytokines can influence de-differentiation in some types of terminally differentiated cells. For example, IL-1β induces chondrocyte de-differentiation (Montaseri et al., 2011; Hong et al., 2014), and some scholars demonstrate that IL-1β also increases vascular smooth muscle cells de-differentiation and proliferation (Sasu and Beasley, 2000; Clement et al., 2006; Zhu et al., 2007). We hypothesized that, in addition to a role in immune responses, IL-1β participates in SC de-differentiation and proliferation. We used a rodent *in vitro* WD model to investigate effects of IL-1β on SC de-differentiation, excluding effects of immune cells and other inflammatory cytokines.

## Materials and Methods

### *In vitro* WD Model

The Ethics Committee for Animal Research at the Ninth People’s Hospital affiliated to Shanghai Jiao Tong University approved all experimental protocols involving the use of rats. Sciatic nerve (SN) explant cultures were performed as previously reported by Thomson et al. (1993) with minor modifications (Thomson et al., 1993; Lee et al., 2009). Eight-week old male Sprague–Dawley rats, obtained from the Ninth People’s Hospital Animal Center, Shanghai, China, were euthanized with 10% chloral hydrate. SNs were exposed and carefully cut, and WD induced by nerve injury. Connective tissues surrounding the SNs and epineurium were carefully detached in DMEM under a stereomicroscope (Carl Zeiss). SNs were dissected into explants 1 cm in length. SN explants were established by loosely separating small bundles of fibers from the isolated nerve. The bundles of nerve were maintained in DMEM containing 10% fetal bovine serum, 100 U/ml penicillin, 100 mg/ml streptomycin and 0.25 mg/ml amphotericin B at 37°C with 5% CO_2._ Cell culture reagents were obtained from Invitrogen. Nerve bundles were treated with or without various concentrations of recombinant rat IL-1β (401-ML-025, R&D Systems).

Schwann cells and fibroblasts compose most of the non-neuronal cell population in intact PNS, whereas macrophages, which are scarce in intact PNS, are recruited in large numbers from the third day after injury (Perry et al., 1987; Reichert et al., 1994). In our modified *in vitro* WD model, in which epineurium were carefully detached, SCs comprise the majority of the cell population in this model and are the primary object of this study.

### RNA Extraction, RT-qPCR, and qPCR Analysis

Total RNA was isolated from SNs in the *in vitro* WD model. Briefly, SNs were washed with PBS and lysed with TRIzol reagent (Invitrogen), according to the manufacturer’s protocol. 2 mg of total RNA was used for reverse transcription (RT), and the products used in qPCR. Target genes were quantified with an ABI 7500 Real-Time PCR System (Life Technologies). PCR primer pairs were designed based on sequences of different exons of the corresponding genes (Table 1). All PCR amplifications were performed with an initial denaturation at 95°C for 10 s, followed by 40 cycles at 95°C, 30 s, 60°C, 30 s, followed by melting curve analysis at 95°C, 60 s and 60°C, 30 s.

**TABLE 1:** List of oligonucleotides used for quantitative real time PCR.

**Target gene**	**Sequence**	**References**
Rat IL-1β	5′ AGT GTG TGA TGT TCC CAT TAG 3′ 5′ GCT TAT GTT CTG TCC ATT GAG 3′	NM_031512.2	
Rat p75NTR	5′ GAG GAT TAC GGA CCT ATC TGA 3′ 5′ TGC CTT TCT CTG GGT TTT AC 3′	NM_012610.2	
Rat MPZ	5′ CAT TGT GGT TTA CAC GGA CAG 3′ 5′ CTT GGC ATA GTG GAA GAT TGA 3′	NM_017027.1	
Rat c-JUN	5′ TGA AGT GAC CGA CTG TTC TAT 3′ 5′ CTT AGG GTT ACT GTA GCC GTA G 3′	NM_021835.3	

### Quantification of IL-1β by ELISA

Fifteen 30 mm SN segments without epineurium were harvested from rats. Three SN segments were frozen immediately in liquid nitrogen. The remainder were cultured as per the *in vitro* WD model. These nerve segments were harvested after 12, 24, 36, and 48 h. Nerve segments were extracted in 1 ml PBS containing a mixture of protease inhibitors (Roche Molecular Biochemicals). We used two-site sandwich ELISA to identify and quantify IL-1β in SNs, according to the manufacturer’s instructions (Duo-Set; R&D Systems). SNs were rinsed in ice-cold PBS to remove excess blood. Tissues were minced and homogenized in 1 ml PBS with a glass tissue grinder on ice. The resulting suspension was subjected to ultrasonication to further disrupt cell membranes. Homogenates were centrifuged for 15 min at 1500 × *g* and supernatants used for ELISA.

### Immunofluorescent Staining

Teased nerve fibers mounted on slides were treated with PBS containing 4% Paraformaldehyde for 30 min and blocked with PBS containing 0.2% Triton X-100 and 2% BSA for 60 min. Nerve fibers were incubated with primary antibody (anti-p75NTR antibody 1:1000, ab52987, Abcam; anti-myelin protein zero (MPZ) antibody, 1:1000, ab31851, Abcam; anti-c-JUN antibody, 1:1000, #9165, Cell Signaling; anti-Ki67 antibody,1:100, ab16667, Abcam) for 16 h at 4°C and washed three times with PBS. Next, slides were incubated with Alexa 549- or 488-conjugated secondary antibody (1:800, Alexa Fluor) for 2 h at room temperature and washed three times with PBS. Finally, slides were incubated with PBS counterstained with 4′6-diamino-2-phenyl indole (DAPI; Vectashield, Vector Laboratories) to visualize nuclei. DAPI staining was used for enumeration and identification of nuclei. The slides were visualized using a 20×/0.50 Plan-Neofluar lens (Carl Zeiss). c-JUN-positive endonuclear cells were counted to analyze percent of c-JUN (+)in three independent experiments. Ki67-positive endonuclear cells were counted to analyze percent of Ki67 (+)in three independent experiments.

### Western Blotting

Proteins were extracted with RIPA lysis buffer containing 1 mM PMSF (Beyotime) and 40 mM protease inhibitor (Roche Molecular Biochemicals). Lysates were cleared by centrifugation at 14,000 rpm for 5 min at 4°C. Protein concentrations were measured using a BCA protein assay kit (Pierce Chemicals) according to the manufacturer’s protocol. Reducing buffer was added to each protein extract and samples heated to 100°C for 5 min. Reduced samples containing equal amounts of protein were resolved by SDS-PAGE and transferred to a nitrocellulose membrane. The membrane was blocked in 5% BSA and probed with anti-p75NTR, anti-MPZ, and anti-c-JUN antibodies. Blots were washed, incubated with horseradish peroxidase-conjugated secondary antibody (Santa Cruz Biotechnology), and developed with ECL Plus (Amersham Biosciences). Filters were stripped and probed with a goat polyclonal antibody against β-actin (Santa Cruz Biotechnology) to assess equivalent protein loading and to normalize protein levels. Protein bands were analyzed using a chemiluminescence kit (Santa Cruz Biotechnology) and visualized using BandScan 5.0 software western immunoblotting detection system.

### AP-1 Activity Assay

The AP-1 activity assay was used to examine AP-1 activity of SCs in the *in vitro* WD model with or without 5 ng/ml at 6, 12, and 24 h. The DNA binding activity of AP-1 was determined using an AP-1 enzyme-linked immunosorbent assay kit essentially as instructed by the manufacture (Active Motif North America). Briefly, samples in each group were lysed in 10 mM HEPES buffer, pH 7.9, containing 10 mM KCl, 0.1 mM EDTA, 0.1 mM EGTA, 1 mM dithiothreitol, and inhibitors of proteases as described above. After the addition of 0.6% (v/v) Nonidet P-40, the samples were incubated for 15 s on ice and then centrifuged at 13,000 × *g* for 30 s at 4°C. The pellet was suspended in the supplied nuclear lysis buffer and centrifuged at 13,000 × *g* for 10 min at 4°C. Nuclear protein (10 μg) was loaded into the 96-wells of an enzyme-linked immunosorbent assay plate pre-coated with an oligonucleotide containing the sequence 5′-TGAGTCAG-3′ and incubated for 60 min at room temperature. Mutated c-JUN oligonucleotides supplied in the kit were used as specificity controls. AP-1 binding to the nucleotide was detected with an anti-phospho-c-JUN antibody and horseradish peroxidase-conjugated secondary antibody followed by colorimetric analysis.

### TUNEL Staining of Apoptosis

Teased nerve fibers mounted on slides were treated with PBS containing 4% Paraformaldehyde for 15 min, discarded with fixative solution and washed with PBS for 3 times. Next, slides were incubated with 0.1% sodium citrate buffer solution and 0.1% Trion × 100 for 2 min on ice. Rinse slides three times with PBS and add TUNEL reaction mixture (Roche Molecular Biochemicals) on nerve fibers for 60 min in the dark. Rinse slides three times with PBS. Finally, slides were incubated with PBS counterstained with 4′6-diamino-2-phenyl indole (DAPI; Vectashield, Vector Laboratories) to visualize nuclei. DAPI staining was used for enumeration and identification of nuclei. The slides were visualized using a 20 × /0.50 Plan-Neofluar lens (Carl Zeiss). The TUNEL labeled red and DAPI labeled blue were positive at the same time as apoptosis.

### Statistical Analysis

Data are expressed as the means ± SE. Each independent experiment was repeated three times. The significance of differences between two independent samples was statistically assessed using Student’s *t*-test. The statistical significance of differences between groups was determined by one-way ANOVA followed by the Least Significant Difference (LSD) test. *p*-values < 0.05 were considered significant.

## Results

### IL-1β mRNA Expression and IL-1β Protein Production by SCs in *in vitro* WD Model

**FIGURE 1:**
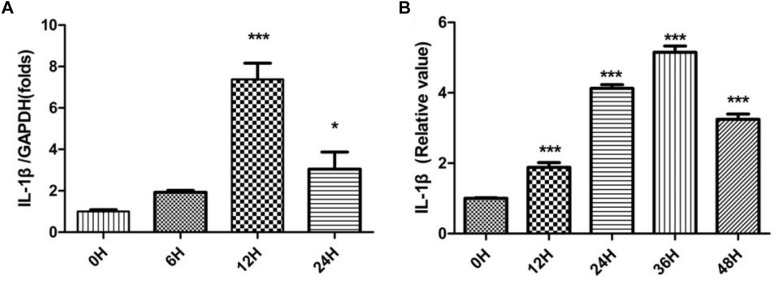
IL-1β mRNA expression and IL-1β protein production. The relative quantification of IL-1β mRNA was performed by qPCR **(A)** and IL-1β protein levels were determined by ELISA **(B)** in SCs. The control group is labeled as 0 h. SCs harvested from SNs at different times were compared to the control group. ^*^p < 0.05, ^∗∗∗^*p* < 0.001.

Sciatic nerves without epineurium were frozen immediately afterremoval from rats, as a control group. SNs were cultured as per *in vitro* WD model and harvested at different times. These SN tissues were used thereafter as sources for the detection of IL-1β mRNA expression and IL-1β protein production by SCs. Relative quantification of IL-1β mRNA was performed by qPCR, and results were analyzed by one-way ANOVA followed by LSD test (Figure 1A). The analysis revealed expression of IL-1β mRNA by SCs increased after SN injury, peaking at 12 h.

IL-1β exerts its biological activity as a soluble molecule only. We further tested for the presence of soluble IL-1β using ELISA. Expression levels of soluble IL-1β by SCs were compared with quantities in SNs. Relative values of IL-1β were analyzed by one-way ANOVA followed by LSD test (Figure 1B). Production of soluble IL-1β protein by SCs increased after nerve damage, peaking at 36 h.

### Effect of IL-1β on De-Differentiation of SCs

**FIGURE 2:**
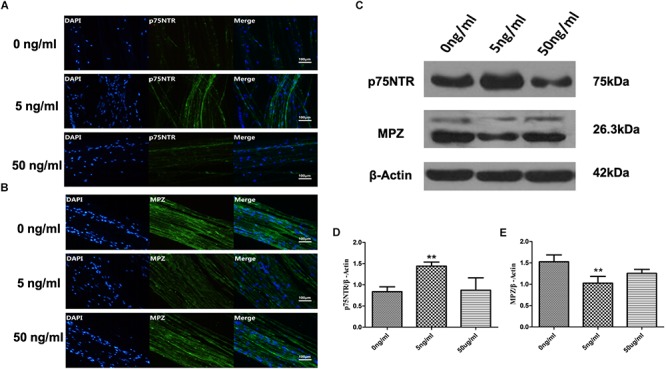
Expression of p75NTR and MPZ in the *in vitro* WD model at 48 h. Immunofluorescent staining of p75nNTR **(A)** and MPZ **(B)**, and western blotting to quantitatively analyze expression levels **(C–E)** in SCs with various concentrations of IL-1β at 48 h. Results are means ± SE (*n* = 3 per group) from independent experiments. ^∗∗^*p* < 0.01.

Sciatic nerve SCs were treated with various concentrations of recombinant rat IL-1β (0, 5, and 50 ng/ml) in this *in vitro* WD model and harvested after 48 h. We detected expression of p75NTR, a marker of SC de-differentiation (Jessen and Mirsky, 2008; Shin et al., 2013), and MPZ, an essential factor in myelination in these SNs to assess SCs de-differentiation (Warner et al., 1998). Immunofluorescent staining demonstrated that expression of p75NTR increased and levels of MPZ decreased in the 5 ng/ml IL-1β group compared with 0 and 50 ng/ml groups (Figures 2A,B) at 48 h. Additionally, the difference of p75NTR and MPZ expression between the 50 and 0 ng/ml IL-1β groups is not obvious. These were quantitatively verified by western blotting, with results analyzed by one-way ANOVA followed by LSD test (Figures 2C–E). 5 ng/ml IL-1β promoted SCs de-differentiation. Nevertheless, a high concentration (50 ng/ml) IL-1β did have the similar effect.

### Effect of IL-1β on Expression of p75NTR and MPZ mRNAs by SCs in *in vitro* WD Model

**FIGURE 3:**
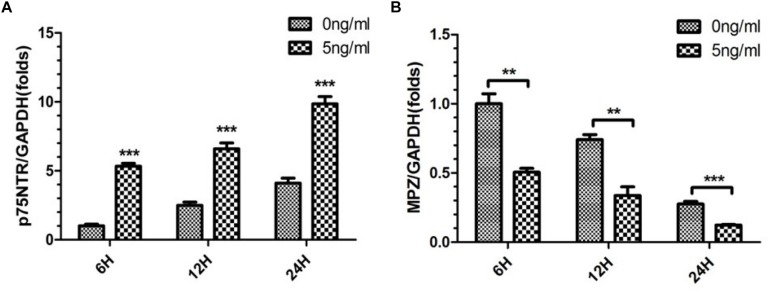
Expression of p75NTR and MPZ mRNA in the *in vitro* WD model. qPCR was used to quantitatively analyze expression of p75NTR **(A)** and MPZ mRNAs **(B)** with or without 5 ng/ml IL-1β at 6, 12, and 24 h. Results are means ± SE (*n* = 3 per group) from independent experiments. ^∗∗^*p* < 0.01, ^∗∗∗^*p* < 0.001.

As 5 ng/ml IL-1β promoted SC de-differentiation, we further quantified expression of p75NTR and MPZ mRNAs by SCs in the present *in vitro* WD model. We harvested SCs from SNs with or without 5 ng/ml IL-1β at 6, 12, and 24 h. qPCR demonstrated that expression of p75NTR mRNA increased in a time-dependent manner in each concentration group, and was elevated more significantly in the 5 ng/ml group compared with the 0 ng/ml group (Figure 3A). Additionally, expression of MPZ mRNA decreased in a temporal manner in both concentration groups, and further decreased in the 5 ng/ml group compared with the 0 ng/ml group (Figure 3B). This suggested that 5 ng/ml IL-1β increased expression of the de-differentiation gene p75ntr and decreased expression of myelination locus MPZ, promoting SCs de-differentiation.

### Effect of IL-1β on c-JUN and AP-1 Activity in SCs

**FIGURE 4:**
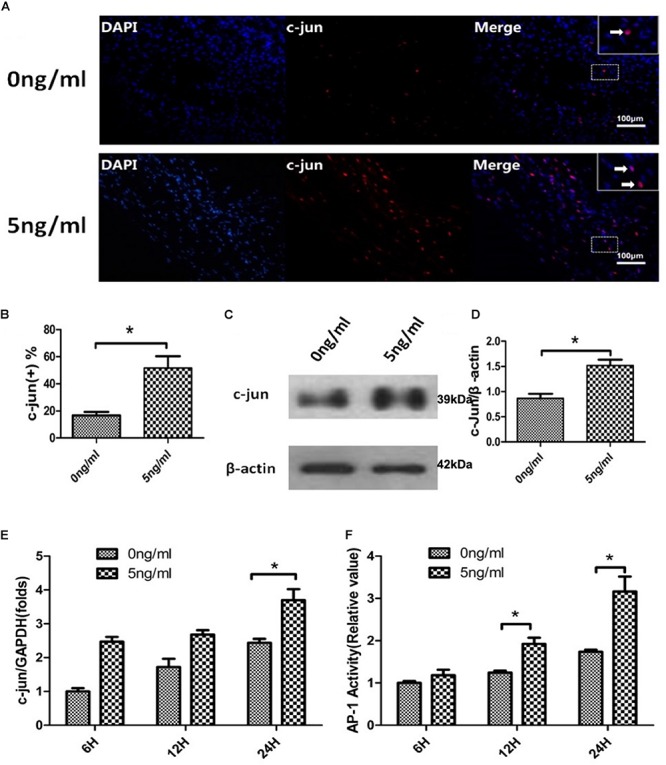
c-JUN and AP-1 activity assay in the *in vitro* WD model. Immunofluorescent staining of c-JUN **(A,** arrows indicate c-JUN(+) cells**)**, analysis of percentage of endonuclear c-JUN(+) cells **(B),** and western blotting to quantitatively analyze expression of c-JUN **(C,D)** in SCs with or without 5 ng/ml IL-1β at 24 h. qPCR was used to quantitatively analyze expression of c-JUN mRNA **(E)**, and AP-1 activity assay to quantitatively analyze activity **(F)** of SCs in the *in vitro* WD model with or without 5 ng/ml IL-1β at 6, 12 and 24 h. Results are means ± SE (*n* = 3 per group) from independent experiments. ^*^p < 0.05.

The transcription factor c-JUN is a global regulator of WD and SCs de-differentiation (Arthur-Farraj et al., 2012; Jessen and Mirsky, 2016) and plays a role in demyelination after PNS injury (Parkinson et al., 2008; Lee et al., 2014). Additionally, these functions of c-JUN were localized in SC nuclei. Therefore, we firstly used immunofluorescent staining to detect the expression of c-JUN in SC nuclei. SCs exposed or not to 5 ng/ml IL-1β were harvested after 24 h. Immunofluorescent staining demonstrated that levels of c-JUN in SC nuclei increased in the 5 ng/ml group compared with the 0 ng/ml group. This was quantitatively verified by percent analysis of endonuclear c-JUN(+) cells (Figures 4A,B). In addition, western blot analysis also revealed that expression of c-JUN in SCs was significantly increased in the 5 ng/ml group in comparison to the 0 ng/ml group (Figures 4C,D). To further verify effects of IL-1β on c-JUN expression, we used qPCR to detect expression of c-JUN mRNA in SCs in our *in vitro* WD model with or without 5 ng/ml IL-1β at 6, 12, and 24 h. qPCR demonstrated that expression of c-JUN mRNA increased in a time-dependent manner in both concentration groups, and increased more significantly in the 5 ng/ml group compared with the 0 ng/ml group at 24 h (Figure 4E). These results suggested that 5 ng/ml IL-1β increased expression of c-JUN and c-JUN transcription factor, and elevated the proportion of endonuclear c-JUN(+) cells in SCs.

The transcription factor c-JUN is a component of the heterodimeric AP-1 transcription factor complex and c-JUN/AP-1 are highly expressed in response to neuronal trauma (Raivich et al., 2004). We therefore examined AP-1 activity of SCs in the *in vitro* WD model with or without 5 ng/ml IL-1β at 6, 12, and 24 h using an AP-1 activity assay. This indicated that AP-1 activity increased in a time-dependent manner in both concentration groups, and was more significantly increased at 12 and 24 h in the 5 ng/ml group compared with the non-exposed group (Figure 4F). AP-1 activity assay results, combined with analyses of c-JUN, suggested that 5 ng/ml IL-1β promoted endonuclear c-JUN expression and stimulation of AP-1 activity in our *in vitro* WD model.

### Effect of IL-1β on Proliferation and Apoptosis of SCs

**FIGURE 5:**
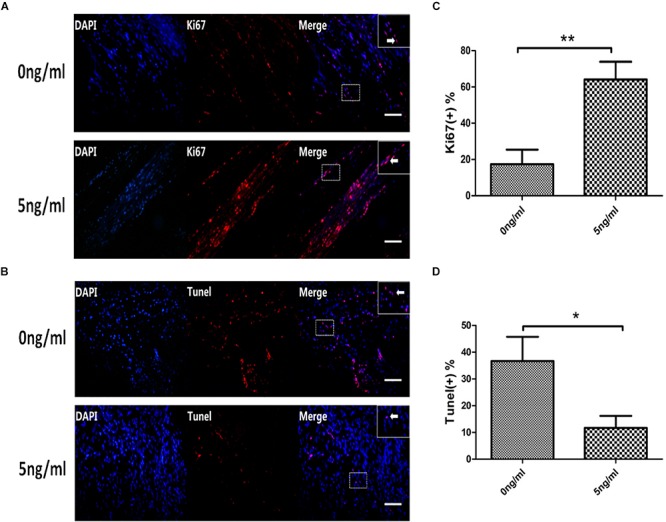
Expression of Ki67 and TUNEL in the *in vitro* WD model. Immunofluorescent staining of Ki67 **(A**, arrows indicate Ki67(+) cells**)**, analysis of percentage of endonuclear Ki67(+) cells **(C)** in SCs with or without 5 ng/ml IL-1β at 48 h. TUNEL staining of apoptosis cells (**B**, arrows indicate apoptosis cells),analysis of percentage of TUNEL(+) cells **(D)** in SCs in the *in vitro* WD model with or without 5 ng/ml IL-1β at 48 h. Results are means ± SE (*n* = 3 per group) from independent experiments. ^*^p < 0.05, ^∗∗^*p* < 0.01.

We harvested SCs from SNs with or without 5 ng/ml IL-1β after24 h. Then, we firstly used immunofluorescent staining to detect the expression of Ki67 in SC nuclei. This demonstrated that levels of Ki67 in SCs nuclei increased in the 5 ng/ml group with the 0 ng/ml group. This was quantitatively verified by percent analysis of endonuclear Ki67(+) cells, and the percent was increased in the 5 ng/ml group compared with the 0 ng/ml group at 48 h (Figures 5A,C). After that, we used TUNEL to detect the percentage of SCs apoptosis. SCs harvested from *in vitro* WD model treated with or without 5 ng/ml IL-1β after 48 h. TUNEL demonstrated that percentage of SCs apoptosis decreased in the 5 ng/ml group compared with the 0 ng/ml group (Figures 5B,D).

## Discussion

In a reminiscent process of the injury responses of zebrafish cardiomyocytes or pigment cells of the newt iris, nerve injury and loss of axonal contact causes mammalian SCs to lose their differentiated morphology, down-regulate myelin gene expression, up-regulate markers of immature SCs, and re-enter the cell cycle (Arthur-Farraj et al., 2012). During WD, SCs de-differentiate and up-regulate genes implicated in promoting axon growth, neuronal survival, and macrophage invasion, and break down their myelin sheaths by autophagy and phagocytosis, and morphologically transform into cells with long, parallel neural processes (Chen et al., 2007; Vargas and Barres, 2007; Gordon et al., 2009; Brosius et al., 2017). SCs de-differentiation allows them to form uninterrupted regeneration tracks (Bands of Büngner) that guide axons back to their targets, and provides a permissive environment for nerve regeneration (Vargas and Barres, 2007; Allodi et al., 2012). Although WD responses, including those resulting from SC injury, are key to damage repair, the molecular mechanisms that control these processes has remained uncertain.

Here, our research showed that the expression of IL-1β mRNA and protein increased in SCs after nerve injury, with IL-1β mRNA peaking at 12 h and IL-1β protein maximal by 36 h in this *in vitro* WD model. These results of our study are similar to previous reports for *in vivo* WD.

To test the hypothesis that, in addition to a role in immune responses, IL-1β participates in SC de-differentiation, we used the *in vitro* WD model treated with various concentrations of IL-1β to investigate effects on SC de-differentiation. Our study suggested that a specific concentration of IL-1β (5 ng/ml) increased expression of p75NTR and decreased levels of MPZ. Nevertheless, a high concentration (50 ng/ml) IL-1β did have the similar effect. SCs in immature states re-express p75NTR, which is a marker of SC de-differentiation (Jessen and Mirsky, 2008; Shin et al., 2013). During de-differentiation, SCs cease to express myelin genes, including MPZ (Warner et al., 1998; Jang et al., 2017). Up-regulation of p75NTR and down-regulation of MPZ have been implicated in SCs de-differentiation and regeneration. Thus, our study suggested that an appropriate concentration of IL-1β promoted SCs de-differentiation and regeneration in WD. On the contrary, a too high concentration of IL-1β might deactivate SCs and could not promote SCs de-differentiation and regeneration in WD.

Previous studies indicate that IL-1β causes de-differentiation of primary cultured articular chondrocytes via the c-JUN/AP-1 pathway (Hwang et al., 2005). On the other hand, IL-1β can induce differentiation of precursor cells. For example, IL-1β is an essential factor for maturation of endothelial precursor cells to ECs (Voronov et al., 2014). However, our research is the first to report that IL-1β promotes SCs de-differentiation in WD. At the same time, there are some reports indicating that IL-1β promotes neurite outgrowth by deactivating RhoA via the p38 MAPK pathway (Temporin et al., 2008a) and sensory nerve regeneration after SN injury (Temporin et al., 2008b). Before and after macrophage recruitment, WD can be defined as two phases characterized by cytokine protein production profiles. The first phase is characterized by the synthesis of IL-1β and other inflammatory cytokines (such as TNF-α, IL-1α, GM-CSF and IL-6). The second phase is characterized by the production of Il-10, Il-6, and a GM-CSF inhibitor molecule, and furthermore, by the diminished production of IL-1β. Therefore, the first phase is largely inflammatory and the second is predominantly anti-inflammatory (Rotshenker, 2011). Thus, based on our research and previous reports, we interpret that appropriate concentrations of IL-1β are conductive to de-differentiation and regeneration of SCs during the first phase of WD.

The transcription factor c-JUN is a key regulator of WD, governs major aspects of injury response, determines the expression of trophic factors, adhesion molecules, the formation of regeneration tracks and myelin clearance and controls the distinctive regenerative potential of peripheral nerves. A key function of c-JUN is the activation of a repair program in SCs and the creation of a cell specialized to support regeneration (Arthur-Farraj et al., 2012; Boerboom et al., 2017). In our study, immunofluorescent microscopy revealed that expressed c-JUN was localized mainly in SC nuclei, consistent with its function as a component of the AP-1 transcription factor. In addition, the endonuclear c-JUN(+) cells fraction of IL-1β (5 ng/ml) treated group was significantly higher than ration of control group. Furthermore, expression of c-JUN protein and mRNA each significantly increased in IL-1β treated cells compared to control cells. Therefore, in our *in vitro* WD model, appropriate concentrations of IL-1β mainly increased expression of c-JUN in SCs nucleus, and promoted de-differentiation and regeneration of SCs.

Among potential intracellular activators of c-JUN is the AP-1 transcription complex, of which c-JUN is a key component (Arthur-Farraj et al., 2012). In this study, we analyzed AP-1 activity and found that IL-1β also promoted activation of SC AP-1 in our *in vitro* WD model. Thus, our findings further reinforce that appropriate concentrations of IL-1β promote de-differentiation and regeneration of SCs via the c-JUN/AP-1 pathway during the first phase of WD. The c-JUN/AP-1 complex is a prominent downstream nuclear target of ERK, JNK and PI3/AKT pathways (Eriksson et al., 2007; North et al., 2010; Mruthyunjaya et al., 2011). Further studies are required to verify whether IL-1β regulates c-JUN/AP-1 via the ERK, JNK and PI3/AKT pathways or others.

Ki67 is a nuclear antigen that acts as a specific and sensitive marker of cell proliferation, and it is a reliable indicator for detecting cell proliferation. Ki67 was not expressed in G_0_ phase and G_1_ early, and began to express in the middle and late G_1_. It was located in the peri-nuclear region, and the expression in S phase and G_2_ phase increased gradually, and M phase peaked (Miller et al., 2018). Therefore, Ki67 is also widely used for pathological evaluation of the proliferation and differentiation of different tumor cells (Berlin et al., 2017). Through Immunofluorescent staining of Ki67 and analysis of percentage of endonuclear Ki67(+) cells in SCs with or without 5 ng/ml IL-1β at 48 h, we found that the appropriate concentration of IL-1β(5 ng/ml) increased the expression of Ki67 in SCs, and increased the positive rate of Ki67. It can be seen that IL-1β can promote the proliferation of SCs during the process of WD. Furthermore, we used TUNEL to detect and quantify the apoptosis rate in SCs, our study suggested that a specific concentration of IL-1β (5 ng/ml) inhibited apoptosis in SCs during WD.

## Conclusion

Schwann cells of WD produce IL-1β which promotes SCs de-differentiation and regeneration via the c -JUN/AP-1 signaling pathway. SCs of WD produce IL-1β which promotes SCs proliferation and induces inhibition of SCs. The precise molecular mechanisms of IL-1β regulation of c-JUN/AP-1 activity are not fully understood, and further studies are required.

## Ethics Statement

The study was approved by the Ethics Committee for Animal Research at Shanghai Ninth People’s Hospital affiliatedto Shanghai JiaoTong University, School of Medicine. In the meantime, in accordance with the approved institutional guidelines and regulations.

## Author Contributions

GC conducted this project and wrote the manuscript. XL executed the experiments. WnW and YW participated in data analysis. FZ conceived the plan. WiW initiated this project and proposed the fundamental frame of this project. All authors read and approved the final manuscript.

## Conflict of Interest Statement

The authors declare that the research was conducted in the absence of any commercial or financial relationships that could be construed as a potential conflict of interest.

## Abbreviations

DAPI: 4′6-diamino-2-phenyl indole
PNS: peripheral nervous system
RT: reverse transcription
SCs: Schwann cells
SNs: sciatic nerves
WD: Wallerian degeneration.
